# Impact of Seasons and Dioecy on Therapeutic Phytoconstituents of *Tinospora cordifolia*, a Rasayana Drug

**DOI:** 10.1155/2014/902138

**Published:** 2014-08-10

**Authors:** Namrta Choudhry, Shweta Singh, Mohammad Badruzzaman Siddiqui, Sayyada Khatoon

**Affiliations:** ^1^Department of Botany, Aligarh Muslim University, Aligarh 202002, India; ^2^Pharmacognosy and Ethnopharmacology Division, CSIR-National Botanical Research Institute, Lucknow 226001, India

## Abstract

*Tinospora cordifolia* (Thunb.) Miers, Menispermaceae, is a dioecious creeper, commonly known as “Giloe” or “Guduchi” with significant medicinal importance in the traditional systems of medicine. It is designated as Rasayana drug in Ayurveda and recommended for a number of diseases and also as adaptogen and immunomodulator. The safety and efficacy of herbal medicines are closely correlated with the quality of the source materials. The aim of this study is to see the effect of seasons on phytoconstituents and how these vary in male and female stem samples of *T. cordifolia*. The study revealed that total phenolics and total sugar concentration obtained highest values in summer season while starch and tannin content were found maximum in winter season in both the genders. However, biomarkers, tinosporaside and berberine, reached to their highest concentration in monsoon season. Further, antioxidant potential revealed the highest inhibition percentage in winter season as well as in late summer season. The results of this study suggest that the female plant is best for its therapeutic phytoconstituents and the best harvesting seasons may be either winter or late summer for antioxidant potential and immunomodulator activities and monsoon for antidiabetic activity of *T. cordifolia*.

## 1. Introduction

Maximum production of metabolites depends on age and growth phase of the plant. Harvesting of crude drugs with higher concentration of active principle is prerequisite in preparation of efficacious drugs [1]. Season had its impact on quantity and/or quality of active principles and secondary metabolites in medicinal plants [2]. The biosynthesis of secondary metabolites although controlled by genetic factors is affected by a number of reasons, that is, environmental factors, developmental stages of the plants, function, and activities of different plant parts. These will result in fluctuations in the concentration and quantities of secondary metabolites throughout the year and growing stages of the plants. It is generally assumed that the material is best collected when the organ in question has reached its optimal state of development; based on such assumptions, herbs are collected at the flowering stage [3].

Secondly, the phenomenon of dioecy in plants is rarer and is confined to about 7% of the known taxa, widely distributed in different orders and families [4]. Though human awareness of this phenomenon is as old as from the Babylonian times (ca. 2300BC) where different sexes were known for date palms [5], however, its importance in use of plant material in traditional medicine seems to have escaped the attention of ancient masters as well as of the present day scientists as is evident from lack of references in global literature on traditional medicine. It is notable that nearly twenty-five plant species with known dioecism are presently being used traditionally. However, the gender-specific responses are influenced by environmental conditions [6] such as differences in growth rates [7] and in secondary metabolites [8] are reported in plants.

*Tinospora cordifolia* (Thunb.) Miers (*Guduchi*) is an important dioecious plant, which belongs to the family Menispermaceae. In Hindi, it is known as Giloe [9] which is a Hindu mythological term that refers to the heavenly elixir that has saved celestial beings from old age and kept them eternally young. In Ayurveda, it is designated as* Rasayana* drug recommended to enhance general body resistance and promote longevity and as antistress and adaptogen [10, 11]. The fact that it is called “Amrita” signifies its use for revitalization and its importance in Ayurveda. This significant plant is also mentioned in important Pharmacopoeias [12]. Several reports on its chemical constituents, medicinal properties, and validation of therapeutic claims have already been published [13–15].* T. cordifolia* is reported for adaptogenic, anticancer, anti-inflammatory, antiallergic, antidiabetic, antioxidant, antipyretic, hepatoprotective, immunomodulator, and diuretic activities and is also found useful for the protection against the toxicity of cancer chemotherapy [14]. Major constituents, namely, alkaloids, cardiac glycosides, flavonoids, lignans, saponins, steroids, tannins, terpenoides, and so forth [13, 15] are reported from* T. cordifolia*. Berberine is an isoquinoline alkaloid reported to have anticancer [16, 17], antidiabetic [18], and immunomodulatory [19, 20] properties. Starch of* T. cordifolia*, commonly known as “Giloe Satva,” is reported as immunomodulator [18, 21, 22] and antimetastatic [23]. Tinosporaside is reported for antihyperglycemic activity [24].

Numerous reports had been published on active chemical constituents and several biological activities [25, 26] of* T. cordifolia* without taking into consideration the seasonal and gender impacts. Hence, the aim of this study is to determine the phytoconstituents with respect to different seasons and genders to ascertain the best gender and time to harvest this important medicinal plant.

## 2. Materials and Methods

### 2.1. Selection of Seasons and Collection of Plant Materials

The Aligarh district located in Uttar Pradesh experiences tropical monsoon type climate of the Great Gangetic Plain of northern India [27] having the year divided into winter (November–February) and summer (March–October) the latter is again subdivided into early summer (March–May), late summer (June-July), rainy season (August-September), and the season of the retreating of Monsoon (October–November) [28]. Male and female plants of* Tinospora cordifolia* were collected during 2010–2012 in the months of January, April, June, August, and October for studying seasonal as well as dioecy variation. The plant materials were identified by the taxonomist Dr. M. B. Siddiqui and vouchers plant specimen of* T. cordifolia* No. A.M.U/31337 (male), 31338 (female)/2010 were deposited in Botany Department, Aligarh Muslim University, Aligarh for future reference. Monthly mean maximum and mean minimum temperature and relative humidity were selected for this study [29].

### 2.2. Sample Preparation

The collected plant materials were shade dried for a day and then dried completely in an oven at 40°C. The plant materials were coarsely powdered using a rotary grinder and stored at 25°C airtight containers and powdered to 60 mesh when required for quantification of phytochemicals, antioxidant, and HPTLC analysis. Powdered dried plant samples (10 g) were extracted with methanol (4 × 25 mL, three times each for 3 days). The extracts were combined (separately for each samples), filtered, evaporated to dryness using a rotary evaporator, and then lyophilized. Accurately weighed extract (10 mg) was dissolved in 1 mL of methanol and filtered through a 0.45 *μ*m filter membrane; the filtrate was used as sample solution. Standard solution of tinosporaside (1.0 mg/mL) and berberine (0.1 mg/mL) as reference biomarkers was also prepared in methanol. All chemicals used were of analytical grade. Berberine standard was procured from Sigma-Aldrich and tinosporaside from Dr. Rakesh Maurya, Scientist, CSIR-Central Drug Research Institute, Lucknow. All chemicals used were of analytical grade.

### 2.3. Estimation of Starch Content

The extract (10% (*m/v*)) was prepared in 80% (*v/v*) ethanol. Samples were centrifuged at 2000 ×g for 15 minutes. 4 mL of distilled water was added to the residue obtained which was heated on a water bath for 15 minutes and macerated with the help of glass rod. 3 mL of 52 percent perchloric acid was added in each sample and again centrifuged at 2000 ×g for 15 minutes. The supernatant obtained is made up to known volume (generally up to 10 mL or depending on the expected concentration of starch). 0.2 mL aliquot, added into 0.1 mL of 80% (*v/v*) phenol and 5 mL  conc. sulphuric acid, and made the volume up to 10 mL with 80% (*v/v*) ethanol, cooled in ice bath. Total sugar was calculated as D-glucose (mg/mL) by using the following equation based on the calibration curve, *y* = 22.5*x* − 0.041, *r* ^2^ = 0.964 at 490 nm using UV-1 double beam spectrophotometer (Thermo Electron Corporation, Cambridge, England), where *y* was the absorbance and *x* the D-glucose equivalent (mg/mL). Three replicates were determined for each plant part of all seasons [30].

### 2.4. Estimation of Sugar Content

0.5 gram powdered material was homogenated in 80% (*v/v*) ethanol with the help of centrifuge at 2000 ×g for 15 minutes. The supernatant obtained is made up to known volume (generally up to 10 mL or depending on the expected concentration of sugar). 0.2 mL aliquot, added into 0.1 mL of 80% (*v/v*) phenol and 5 mLconc. sulphuric acid, and made the volume up to 10 mL with 80% (*v/v*) ethanol and cooled in ice bath. Total sugar was calculated as D-glucose (mg/mL) by using the following equation based on the calibration curve, *y* = 23.3*x* − 0.057, *r* ^2^ = 0.954 at 490 nm using UV-1 double beam spectrophotometer, where *y* was the absorbance and *x* the D-glucose equivalent (mg/mL). Three replicates were determined for each plant part of all seasons [30].

### 2.5. Estimation of Total Phenolic Content

Stock solution (1 mg/mL) of extract was prepared in methanol. From the stock solution suitable quantity of the extract was taken into 25 mL volumetric flask, in which 10 mL of water and 1.5 mL of Folin and Ciocalteu's reagent were added and mixture was kept for 5 min and then added 4 mL of 20% Na_2_CO_3_ and made up to 25 mL with distilled water. Mixture was kept for 30 min and absorbance recorded at 765 nm. Total phenolic content was calculated as gallic acid (mg/mL) using the following equation based on the calibration curve: *y* = 118.0*x* + 0.069, *r* ^2^ = 0.999, where *y* was the absorbance and *x* was the gallic acid equivalent (mg/mL). Three replicates were determined for each plant part of all seasons [31].

### 2.6. Estimation of Total Tannin Content

2 g powdered plant material was extracted with 100 mL distilled water by boiling on water bath for 6–8 hrs, filtered and made up the volume to 100 mL in the volumetric flask. 5 mL Folin and Ciocalteu's reagent and 10 mL saturated sodium carbonate were added to 1 mL aliquot of it and made the volume up to 100 mL in volumetric flask. The instrument was calibrated through blank and took the corresponding absorbance of different samples, and tannin content was calculated by the following equation based on the calibration curve: *y* = 77.76*x* + 0.085, *r* ^2^ = 0.999 at 760 nm, using UV-1 double beam spectrophotometer, where *y* was the absorbance and *x* the tannic acid equivalent (mg/mL). Three replicates were determined for each plant part of all seasons [32].

### 2.7. Estimation of Tinosporaside and Berberine through High Performance Thin Layer Chromatography

HPTLC was performed on 10 cm × 20 cm Higlachrosep plates coated with 0.2 mm layers of nanosilica containing UV 254 fluorescent indicator (S.D. Fine Chemicals, India). Samples (20 *μ*L) were applied as bands 6 mm wide, 11.3 mm apart, 10 mm from the bottom edge, starting 15 mm from the edge of the plate, and by means of a (CAMAG, Switzerland) Linomat applicator fitted with a Hamilton syringe (100 *μ*L). Standards of the markers tinosporaside and berberine were also applied to the plates. The plates were developed to a distance of 8.0 cm with 20 mL chloroform : methanol : water (8 : 2 : 0.2* v/v/v*) as mobile phase, in a CAMAG twin-trough chamber previously saturated with mobile phase vapour for 30 min at 24°C. After removal from the chamber, plates were completely dried in air at room temperature (24°C). Densitometric scanning at 220 nm for tinosporaside and at 320 nm for berberine was performed with a CAMAG TLC scanner III with winCATS 3.2.1 software. Photographs were taken by means of a CAMAG Reprostar 3 video documentation unit by illumination at UV366 nm and under visible light after derivatization with anisaldehyde sulphuric acid reagent [33].

### 2.8. Antioxidant Activity

#### 2.8.1. 2, 2′-Diphenyl-1-picrylhydrazyl (DPPH) Radical-Scavenging Assay

To evaluate antioxidant activity, solution of 0.135 mM DPPH in methanol was prepared and 1.0 mL of this solution was mixed with 1.0 mL of extract in methanol containing 0.02–0.1 mg of the extract. The reaction mixture was vortexed thoroughly and left in the dark at room temperature for 30 min. The absorbance of the mixture was measured at 517 nm using UV-1 double beam spectrophotometer. Ascorbic acid was used as reference standard. The ability to scavenge DPPH radical was calculated by the following equation: DPPH radical scavenging activity (%) = [(Abs control − Abs sample)/(Abs control)] × 100, where Abs control is the absorbance of DPPH radical + methanol; Abs sample is the absorbance of DPPH radical + sample extract/standard [34].

#### 2.8.2. *β*-Carotene Bleaching (BCB) Assay

The antioxidant activity (AOA) of the different samples was evaluated using the *β*-carotene bleaching assay following the method of Amarowicz et al. [35]. In brief, a solution of *β*-carotene was prepared by dissolving 2 mg of *β*-carotene in 10 mL of chloroform and 2 mL of this solution was pipette into a 100 mL round-bottom flask. After chloroform was removed under vacuum, using a rotary evaporator at 40°C, 40 mg of purified linoleic acid, 400 mg of Tween 40 as an emulsifier, and 100 mL of aerated distilled water were added to the flask with vigorous shaking. Aliquots (4.8 mL) of this emulsion were transferred into a series of tubes containing 200 *μ*L of the extract (200 ppm in methanol). The total volume of the system was adjusted to 5 mL with methanol. As soon as the emulsion was added to each tube, the zero time absorbance was measured at 470 nm with a UV-1 double beam spectrophotometer. Subsequent absorbance readings were recorded by keeping the samples in a water bath at 50°C. Blank samples, devoid of *β*-carotene, were prepared for background subtraction.

### 2.9. Statistical Analysis

All values reported in this work are means of three independent determinations. The mean values ± SD are given in graphs. All the data has been statistically analyzed by one way analysis of variance (ANOVA) in randomized complete block design (RCBD) to check the variability of data and validity of results. Differences at *P* < 0.05 were considered statistically significant. Comparison between means was done by LSD test [36].

## 3. Results

### 3.1. Effect of Season and Gender on Total Sugar, Starch, Phenolics, and Tannin Contents

**Figure 1:**
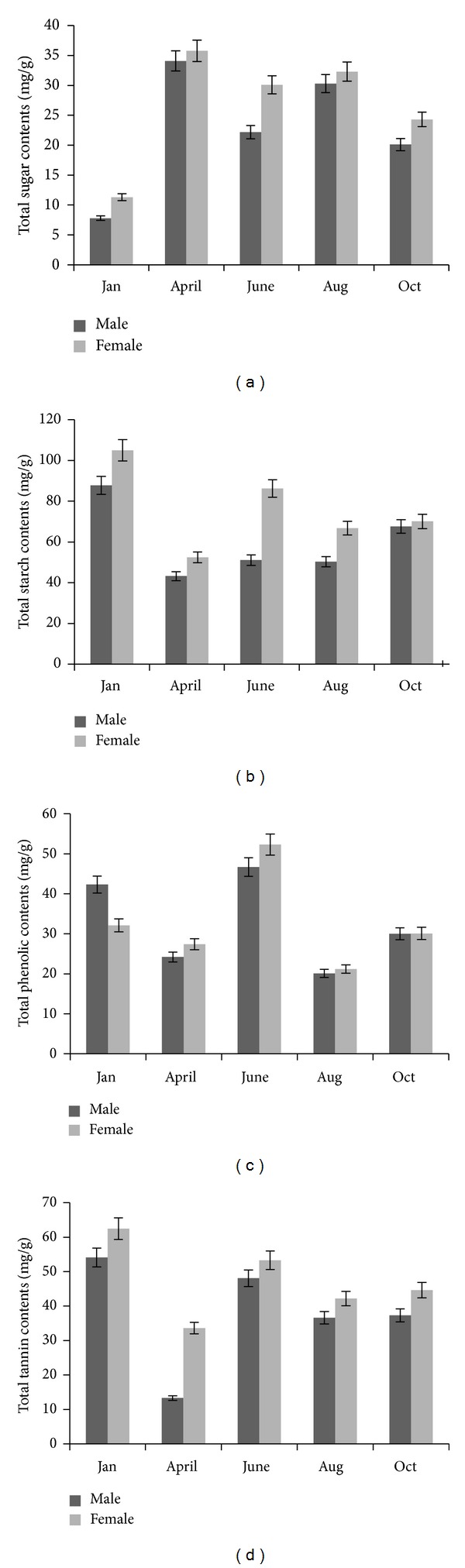
Effect of different seasons on concentration. (a) Total sugar content, (b) total starch content, (c) total phenolic content, and (d) total tannin content. Mean ± SD (*n* = 3). ANOVA, *P* < 0.05.

The sugar concentration varied significantly (ANOVA, *P* < 0.05) in different seasons. Quantification was done for carbohydrate in which total sugar content showed highest percentage in the early summer samples 34.1 mg/g in male and 35.8 mg/g in female with fluctuating trends in other seasons (Figure 1(a)). Furthermore, female samples showed significantly higher value than male (ANOVA, *P* < 0.05).

Likewise total starch content showed significantly higher (ANOVA, *P* < 0.05) value in winter season 87.8 mg/g in male and 105.0 mg/g in female and gets reduced up to minimum value in early summer season 43.2 mg/g in male and 52.5 mg/g in female (Figure 1(b)). Moreover, female samples found significantly (ANOVA, *P* < 0.05) higher concentration of starch content comparatively to male samples. Sugar and starch showed vice versa in concentration.

Analysis of total phenolic concentration in different seasons was found significantly effected in all seasons (ANOVA, *P* < 0.05). Maximum concentration of phenolic content was obtained in late summer season 46.7 mg/g in male and 53.2 mg/g in female with reduced concentration up to minimum level 20.1 mg/g in male and 21.2 mg/g in female in monsoon season (Figure 1(c)). Total tannin content significantly (ANOVA, *P* < 0.05) increased during winter season 54.1 mg/g in male and 62.5 mg/g in female. The concentration significantly (ANOVA, *P* < 0.05) gets reduced to minimum in early summer season in both genders 13.3 mg/g in male and 33.6 mg/g in female (Figure 1(d)). Female samples found significantly (ANOVA, *P* < 0.05) higher concentration of total phenolics and total tannin content when compared with male samples.

### 3.2. Effect of Season and Gender on Tinosporaside and Berberine Content

**Figure 2:**
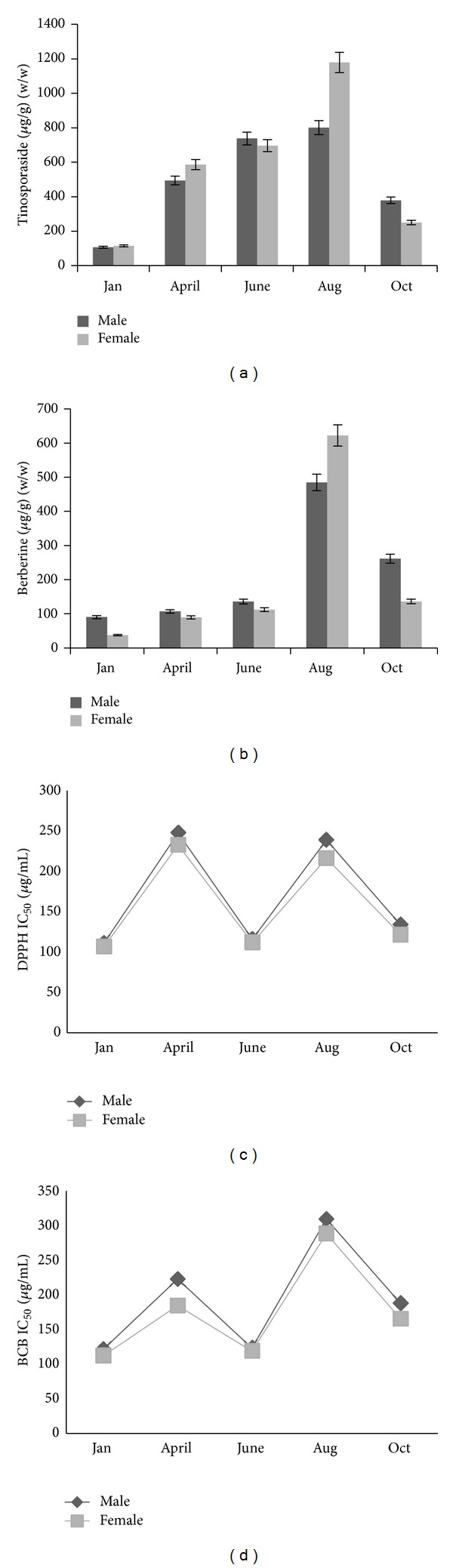
Effect of different seasons on concentration and antioxidant potential. (a) Tinosporaside, (b) berberine, (c) DPPH, and (d) BCB. Mean ± SD (*n* = 3). ANOVA, *P* < 0.05.

**Figure 3:**
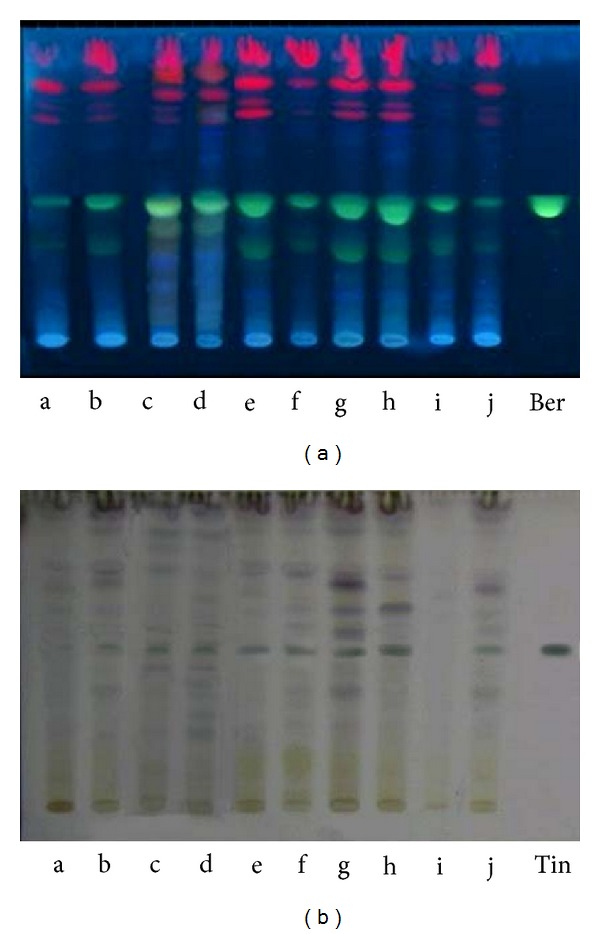
HPTLC fingerprint profile of methanolic extracts of male and female stem of* Tinospora cordifolia* collected in different seasons along with biomarkers, tinosporaside and berberine. a, c, e, g, i: male samples; b, d, f, h, j: female samples (a-b: collected in January; c-d: collected in April; e-f: collected in June; g-h: collected in August; i-j: collected in October); Ber: berberine; Tin: tinosporaside. (a) Documentation under UV 366 nm and (b) documentation under visible light after derivatization with anisaldehyde sulphuric acid reagent.

**Figure 4:**
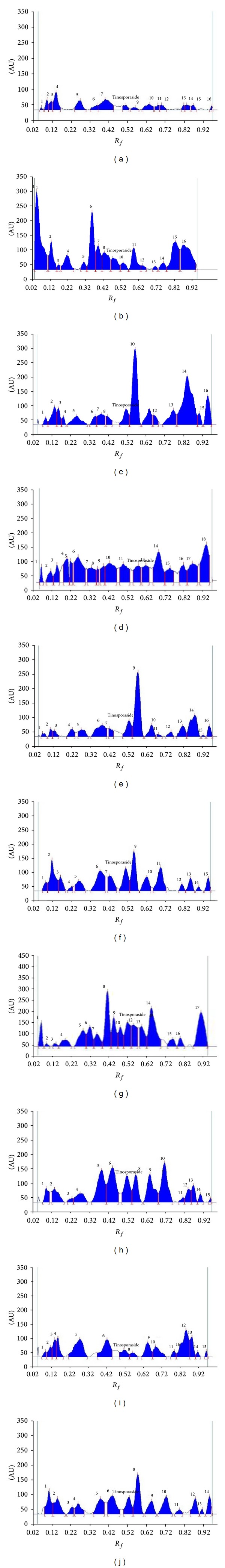
HPTLC densitometric scanning at 220 nm of methanolic extracts of male and female stem of* Tinospora cordifolia* collected in different seasons along with biomarker tinosporaside. a, c, e, g, and i: male samples; b, d, f, h, and j: female samples (a-b: collected in January; c-d: collected in April; e-f: collected in June; g-h: collected in August; i-j: collected in October).

**Figure 5:**
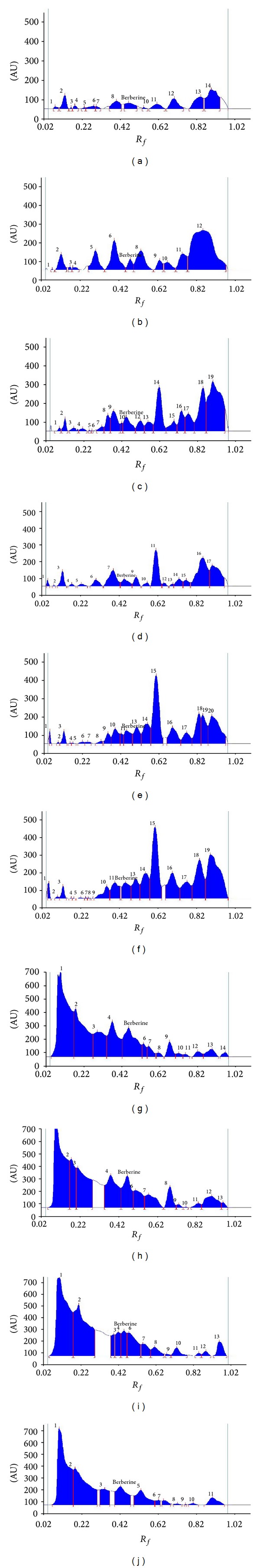
HPTLC densitometric scanning at 320 nm of methanolic extracts of male and female stem of* Tinospora cordifolia* collected in different seasons along with biomarker berberine. (a, c, e, g, i): male samples; (b, d, f, h, j): female samples ((a-b): collected in January; (c-d): collected in April; (e-f): collected in June; (g-h): collected in August; (i-j): collected in October).

In the present study, significant (ANOVA, *P* < 0.05) effect of season was observed on tinosporaside concentration which decreased during winter, 107 *μ*g/g (*w/w*) in male, and 115 *μ*g/g (*w/w*) in female which reached highest in monsoon season, that is, 800 *μ*g/g (*w/w*) in male and 1178 *μ*g/g (*w/w*) in female (Figures 2(a), 3(b), and 4). Likewise, in concentration of berberine significant (ANOVA, *P* < 0.05) fluctuation was observed in each season that is minimum value during winter, that is, 90 *μ*g/g (*w/w*) in male and 37 *μ*g/g (*w/w*) in female; however, increase in concentration was noticed in monsoon season which was 484 *μ*g/g (*w/w*) in male and 622 *μ*g/g (*w/w*) in female (Figures 2(b), 3(a), and 5). Female samples showed significantly (ANOVA, *P* < 0.05) higher concentration of tinosporaside and berberine content, that is, 47 *μ*g/g (*w/w*) and 28 *μ*g/g (*w/w*), respectively, than male samples.

### 3.3. Effect of Season and Gender on Antioxidant Potential

It should be noted that lower IC_50_ value means higher activity. During present study, a significant (ANOVA, *P* < 0.05) increase was observed in winter and late summer season compared to other seasons. Winter and late summer season showed non-significant difference (ANOVA, *P* > 0.05). Lowest IC_50_ value (or the highest antioxidant activity) in this species was observed in winter season 111.5 *μ*g/mL in male and 107.0 *μ*g/mL in female while 116.2 *μ*g/mL in male and in 112.0 *μ*g/mL in female in late summer by DPPH method. However, in case of BCB method 121.5 *μ*g/mL in male and 112.3 *μ*g/mL in female were obtained in winter while 123.4 *μ*g/mL in male and 118.8 *μ*g/mL in female were found in late summer season. Maximum IC_50_ value recorded in early summer 247.7 *μ*g/mL in male and 232.7 *μ*g/mL in female, with DPPH analysis while 309.6 *μ*g/mL in male and 288.5 *μ*g/mL in female in monsoon season with BCB analysis (Figures 2(c) and 2(d)). During the present study, it was observed that in female samples significant (ANOVA, *P* < 0.05) lower value of IC_50_ than that in male samples was recorded during both studied methods.

## 4. Discussion

Value addition of the medicinal plants is very much essential for commercial exploitation as well as the medicinal value of the raw drugs. Even authenticated plant material may not be of desired quality and strength and not conforming to the physicochemical parameters or the concentration of the phytoconstituents or active therapeutic agents as per the pharmacopoeial standards or the consumer/industry requirements. Such material is liable to be rejected or accepted at very low price causing not only economic loss to the cultivators or collectors of the medicinal plants but also doubtful efficacy or the potency of the raw drug in the alleviation of the human suffering. Harvesting of medicinal plant/part with higher concentration of active principle is prerequisite in preparation of efficacious drugs. These need to be very much considered and the collection of the material should be made in the appropriate season. Stem of* T. cordifolia* is a Rasayana drug of Ayurveda and in high demand as medicine for different kinds of illness [10, 11, 13–15]. The significant variations were observed in its phytoconstituents not only in genders but also in different seasons (Figures 1 and 2). Rate of assimilation, translocation, and its utilization indicates the growth potential of plants under prevailing conditions. Period of accumulation, nature, and quality of metabolism in the plants is variable with the season.

Maximum percentages of phenolic compounds in late summer season might be due to increased activity of phenylalanine ammonia-lyase (PAL) under water stress. PAL is an important enzyme in the biogenesis of various phenolic compounds by activation of a number of genes involved in phenylpropanoid pathway reported in many plant species [37]. It was reported that under water stress condition, less protein was used for plant maintenance which result increased the availability of the phenylalanine (Phe) pool; hence, more Phe is available for the production of secondary metabolites [38]. Nonoptimum temperatures affect the expression of genes for main enzymes of phenolic compound (PC) biosynthesis: phenylalanine ammonia-lyase, chalcone synthase, and flavanon-3-hydrolase, and this is reflected in the composition of flavonoids and phenolcarbonic acids in plant tissues [39]. On the contrary, the maximum tannin content in winter correlated for adaptation with frost resistant cells, so as to avoid any injury during unfavourable temperate conditions [40]. Upadhye et al. documented increased tannin compound in water stress as a consequence of low temperature [41].

Starch, a complex carbohydrate, is a polymer of glucose molecules. The synthesis of starch in plant cells begins with the enzyme ADP-glucose pyrophosphorylase (AGPase), which catalyses the reaction of glucose-1-phosphate with ATP to form ADP glucose (liberating pyrophosphate). The ADP glucose then used a substrate by starch synthase enzymes, which add glucose units to the end of a growing polymer chain to build up a starch molecule [42]. The highest accumulation of starch in winter season might be due to the blockage of photoassimilate that could be the adverse temperature impact which was related to the increase of ADP-glucose pyrophosphorylase activity [43]. Krapp and Stitt [44] reported that inhibition of photosynthate export may regulate starch synthesis via changes in gene expression. Second, possible reason of starch accumulation in winter may be the concurrent to freezing tolerance. Mamun et al. [45] showed connection of starch grains increases in chilling-stressed plants which also support our findings in* T. cordifolia*.

More studies have focused on changes in chemical defences during ontogeny including changes in alkaloids [46] and in terpenoids [47]. During the present study course of the growing season, there are often marked fluctuations in tinosporaside and berberine profiles that are highest during active growth phase of this species; that is, from early summer to rainy season this might be due to changes in developmental phases of plant. Similar findings were reported by Cromwell [48] in* Berberis darwinii*, and accumulation of secondary metabolites occurred during developmental stages of plants. Similar results have been reported for other plant species [49, 50]. Reduction was noted in berberine content in female samples; in early summer to late summer season, it could be due to transport of alkaloid in fruits and seeds formation. Similar trends were observed in different species of* Lupinus* at different phenological growth stages [51]. Previous reports of seasonal effect on alkaloids and terpenoid composition are also widely known [52, 53]. Studies have reported both decrease [54] and increase [55] in the production of secondary metabolites as plants develop.

Winter and late summer seasons showed nonsignificant difference (ANOVA, *P* > 0.05) which indicates that plant possesses the highest inhibition percentage in both seasons. High antioxidant capacity reported in winter in* T. cordifolia* might be due to highest concentration of starch which acts as a wonderful immunomodulator. The immunomodulating action of starch (a polysaccharide) was also reported by other workers [56]. On the other hand, highest antioxidant potential in month of June might be due to high concentration of phenolic compounds in respective season. Many studies show total flavonoids, total phenolics, and total tannin correlations with antioxidant properties [57, 58]. Additionally, increase concentration in winter and summer seasons in this study might seem that these seasons subjected to water stress and plant adapted defence strategies by producing antioxidant enzymes against reactive oxygen species (ROS). Many reports indicated that the significant relationship between enhanced antioxidant enzyme activities and increased resistance to environmental stress. Antioxidant enzymes such as superoxide dismutase (SOD), peroxidase (POX), ascorbate peroxidase (APX), and catalase (CAT) were reported to increase under various environmental stresses [59].

It is noted that lower IC_50_ value in female samples showed high antioxidant efficiency. Similarly, phytochemical data also showed female samples, superior in accumulation of secondary metabolites; these sex based differences in phytochemical accumulation and high antioxidant potential might be due to the fact that higher amounts of phytochemicals in female were the part of putative defence function against herbivore attack [60]. Female plants, like nutrient-deficient plants, should display reduced growth, increased carbon nutrient balance in vegetative structures, and higher concentrations of secondary metabolites. Because reduced growth due to their greater reproductive effort may have limited ability to compensate for herbivory relative to male plants [61]. Therefore, female plants have to allocate relatively more resources of defence-related secondary compounds [62]. Various findings concord with our results and showed that female is having higher concentration of metabolites, for instance, tannin and salicortin in* Salix rigida* [61]. Recently, in a comprehensive study recorded gender-based differences in metabolites were also documented [63].

## 5. Conclusion

It is evident from this study that different biochemical attributes varied significantly during different seasons and in genders. The results of gender study suggest that female plant exhibit highest content of total phenolics, total tannin, starch contents, however seasonal study indicate the best harvesting season may be either winter or late summer to obtained optimal yield of phytoconstituents of* T. cordifolia* for its best antioxidant potential and immunomodulator activity. Nevertheless, these compounds appear to be suitable markers of stress in many species, since their levels increase significantly in summer and winter, when plants are subjected to higher or low temperatures and mild drought. Further, female plants can also be harvested during monsoon for the activities related to berberine and tinosporaside such as antidiabetic, anticancer, antitumour, anti-inflammatory, antioxidant, antipyretic, anti-stress, and so forth. The results of this study show that plant ontogeny can influence the accumulation of defence metabolites. We conclude from this research work that temperature stress increased the pharmaceutical and therapeutic importance of the* T. cordifolia* by enhancing the concentration of phytochemicals.
